# The Influence of “Background Theories”: Karl S. Lashley's Epistemology

**DOI:** 10.1002/jhbs.70080

**Published:** 2026-11-12

**Authors:** Kevin Liggieri

**Affiliations:** ^1^ Technical University of Darmstadt Darmstadt Germany

**Keywords:** behaviorism, epistemology, gestalt psychology, ideology, kinesthetic learning, metaphysics, psychology

## Abstract

In this article, my aim is to explore the little‐known epistemology of Karl S. Lashley in the context of the discussions and dynamics between Gestalt psychology and Behaviorism. I will demonstrate how Lashley's position shifted between behaviorism (John B. Watson) and Gestalt and holistic psychology (Wolfgang Köhler, Kurt Goldstein), both in his collaborations and his theories. The article argues that, Lashley did not seek to reconcile behaviorism and Gestalt psychology. Instead, he rejected the need for a unified psychological theory.

## 1 Introduction: Transforming the Battlefield?

Epistemic battlegrounds are common in the history of psychology. Well‐known examples include Ebbinghaus versus Dilthey, James versus Titchener, Small versus Watson, and Freud versus Jung (Baker 2012; Pastore 1974). In the early twentieth century, when debates about learning and behavior intensified, gestalt psychology and behaviorism emerged as the most prominent opposing schools, often seen as mutually exclusive (Ash 1995). Their content and methods appeared polarized: gestalt psychology emphasized “phenomenological” observation, while behaviorism focused on quantitative experimentation (Köhler 1959; Ogden 1933, 151–155; Boring 1930, 308–315; Sokal 1984, 1240–1263; Murray et al. 2000). Gestaltists examined the relationship between individual organisms and their environment. Behaviorists attempted to analyze organisms and control them like machines (Burnham 1966; Mills 1998; O'Donnell 1985; Buckley 1989). Both movements claimed to have the only valid solution for understanding behavior and learning. They often replicated each other's experiments—such as maze learning, puzzle boxes, and kinesthetic learning—but interpreted the results differently (Gruevska 2022; Carroll 2017).

The question whether behaviorists or gestaltists had the adequate definition of learning was frequently discussed by contemporary psychologists and later by historians (Hilgard 1987). Was behaviorism too mechanistic and deterministic? Was gestalt psychology too metaphysical and unscientific? The dispute persisted because neither side would acknowledge its own flaws or theoretical limitations.

Between 1930 and 1950, the psychobiologist Karl S. Lashley approached this theoretical conflict from another perspective. He questioned whether these disputes were truly about theory, or whether they were in fact ideological. Lashley regarded the differences as reflections of distinct worldviews, not as fundamentally different scientific problems. He attempted to change the battlefield.

The central argument of this article is that Lashley increasingly understood the dispute between behaviorism and gestalt psychology as sustained, at least in part, by prior “background theories” that shaped what investigators recognized as relevant evidence. He therefore sought to redefine the problems of learning in neurological terms, to broaden the evidentiary basis on which such problems could be addressed, and to shift attention away from competing comprehensive psychological systems.

My aim is to explore the little‐known epistemology of Karl S. Lashley in the context of the discussions and dynamics between gestalt psychology and behaviorism. I will demonstrate how Lashley's position shifted between behaviorism (John B. Watson) and gestalt and holistic psychology (Wolfgang Köhler, Kurt Goldstein), both in his collaborations and his theories. The paper argues that, unlike Edward C. Tolman, Lashley did not seek to reconcile behaviorism and gestalt psychology (Tolman 1932).

Lashley's rejection of unified psychological theory was not a withdrawal from theoretical questions. Instead, it changed how he approached them. By presenting his work as oriented primarily toward empirical data, Lashley sought to redirect psychology toward neurophysiology as a data‐driven science. Yet, as this article will show, his “data‐driven” epistemology was neither purely empirical nor genuinely theory‐free. Rather, it functioned as an alternative to grand, unified psychological systems whose theoretical assumptions predetermined what could count as evidence. Lashley therefore came to favor an anti‐systematic, anti‐doctrinal, methodologically pluralist, and neurophysiologically constrained empiricism. In this sense, he criticized theories that fixed the meaning of evidence in advance while nevertheless remaining committed to a materialist and organizational account of brain function.

To develop this argument, the article examines four aspects of Lashley's epistemological position. First, how Lashley maintained a materialist monism while rejecting unified or comprehensive psychological systems; second, how he remained skeptical of the theory‐driven selection and interpretation of evidence; third, how he maintained his commitment to materialist and neurophysiological explanations; and fourth, how he reframed psychological problems as questions of neural organization.

The article therefore proceeds in four steps. First, it situates Lashley within the epistemic conflict between behaviorism and gestalt psychology and argues that he did not seek to reconcile the two traditions but instead questioned the comprehensive theoretical systems underlying them. His position combined materialist and experimental commitments with a selective appropriation of holistic and gestalt concepts. Second, the article examines Lashley's critique of closed theoretical systems. It shows how he increasingly argued that experimental evidence was never interpreted independently of prior assumptions, or “background theories,” which shaped the selection, design, and interpretation of experiments. His criticism extended to the limitations of quantification, experimental paradigms, and scientific apparatuses.

Third, the article reconstructs Lashley's methodological alternative: the redefinition of psychological problems as questions of neural organization. Rather than developing another comprehensive theory of learning, he sought to broaden the empirical basis of inquiry by combining behavioral, experimental, and neurophysiological evidence. His materialist monism provided the epistemological framework within which this pluralist empiricism operated.

Finally, the article argues that Lashley did not escape theory through his commitment to data. His rejection of comprehensive psychological systems was itself grounded in a strong materialist conception of the brain. The archival evidence therefore suggests that Lashley's contribution was not the elimination of theory, but a redefinition of how theoretical claims should be constrained by heterogeneous forms of empirical and neurophysiological evidence.

My analysis is based on the relevant research on Lashley's work, his academic networks, and his intellectual biography. Dewsbury (2002a, 2002b) was thus able to clearly demonstrate how Lashley was interpreted through various readings and receptions and, at the same time, presented an alternative to classical behaviorism with his psychobiology. In this context, Weidman's work “Constructing Scientific Psychology: Karl Lashley's Mind‐Brain Debates,” (1999, 2002) deserves special mention because Weidman, on the one hand, highlights Lashley's problematic racist views and, on the other hand—particularly in her book chapter 3, “The Pursuit of a Neutral Science” (48–70)—historizes and analyzes Lashley's opposition to theory. It is precisely this relationship between theory and epistemology that Gruevska also addresses in her discussion of the debate between Lashley and a holistic approach (Gruevska 2022).

Although Lashley's scientific and political (racist) research, his biography, and his connections to behaviorism have already been well studied (Bruce 1986; Dewsbury 2002a, 2002b; Weidman 1999, 2002), I would like to analyze a new and interesting aspect of Lashley's double‐edged epistemology. In this sense, my aim is to expand upon and further differentiate Weidman's innovative research. My concern is not so much to determine whether Lashley was in fact theory‐free, but rather to address the question: “How does his anti‐theory work?” In this sense, Weidman was right to question Lashley's claim to theoretical neutrality, but that does not exhaust the epistemological significance of his rejection of theory. Whereas Weidman was able to demonstrate that Lashley's claim to neutral, fact‐based science is problematic, my article shifts the focus from the social and ideological conditions of his scholarship to the epistemological practices through which Lashley sought to establish empirical authority. Rather than viewing his rejection of theory merely as a contradiction to his own theoretical commitments, I argue that Lashley's anti‐theoretical stance constituted a distinct methodological position. It shaped his understanding of experiments, his selection of evidence, his evaluation of quantitative representations, and his reframing of psychological problems as questions of neural organization.

Lashley did not merely reject psychological theories. As I would like to show, he developed a methodological critique of how theories, experimental paradigms, apparatuses, and forms of representation shape what can appear as evidence in the first place.

Therefore, one of the article's original contributions is to demonstrate that Lashley developed a sophisticated critique of the relationship between background theory, experimental design and selection, apparatus, representation, and the interpretation of evidence. I will show how Lashley reflected on the “history of fashion in experiment” and criticized the ways in which particular experimental paradigms can generate theories that are biased toward the phenomena those paradigms render visible. This becomes apparent, for example, in his critique of the smoothing of learning curves and of the epistemic limitations imposed by quantification.

As Lashley never systematically articulated a theory in a paper or monograph, my analysis of his epistemology relies on unpublished materials (letters, lectures, notes) from his estate.

Karl Spencer Lashley's papers are held at the George A. Smathers Libraries, University of Florida, Gainesville, Florida, and comprise 2.25 linear feet (5 boxes and 1 volume). For my research question, Box 3, “Learning Theory” (folders 13 and 14), and Box 4 “Notes and Drawings: Learning Notes” (Folder 15) are of particular interest.

Folders 13 and 14 from Box 3, “Learning Theory,” were tied together with string. The library dates the manuscripts between 1923 and 1958; however, the majority of the material dates from 1940 to 1958.

Folder 13 comprises 87 pages and is undated. Some of the pages are numbered by Lashley, while others are unnumbered. The notes consist of typescript with handwritten additions and deletions. They also include graphs drawn by Lashley himself and keyword‐style summaries of experiments. Some pages of the manuscript could be a lecture or preparation for a publication. The fact that Lashley writes “footnotes” in one place—indicating that references were to be added here—suggests a later publication.

Lashley provides little clear information regarding the time period. However, quotes such as “The discussion between non‐linearity and linearity theories continued for 20 years” suggest that the texts date to the 1950s. Quotations from Tolman that Lashley uses are also taken from books published in the 1930s (including Tolman's “Purposive Behavior in Animals and Men” [1932]). It is precisely the sporadic references to specific dates—for example, when Lashley writes “which I propose 1929”—that point to a later date probably around 1950. One reference in particular helps narrow down the time frame: Lashley writes a brief introductory CV about Henry W. Nissen, the last entry of which is dated 1947. A dating of the posthumous manuscripts from Folder 13 to around 1950 therefore seems very realistic.

Folder 14 from Box 3 has 159 pages and appears to be a continuation of Folder 13. Folder 14 is also a typescript, again partly without page numbers and containing handwritten additions. However, Lashley has included some organizational headings here, such as “The Prototype of Learning” or “The Significance of Drill.” As in Folder 13, sentences in Folder 14 often simply trail off or consist only of notes. Here, too, the majority of the manuscripts appear to date from the 1940s and 1950s. For example, Lashley refers to Brogden's “Empirical Observations on Dogs” (1942) or to an article by Ritchie, Ebeling, and Roth, “Evidence for continuity in the discrimination of vertical and horizontal patterns,” in the Journal of Comparative and Physiological Psychology from 1950. In some cases, notes can also be found that constitute a commentary on a research paper or a draft letter to Clark L. Hull, though without a date. Overall, Folder 14 appears more coherent and better organized as a body of text than Folder 13. Coherent arguments and chapters can be identified. This could indicate a lecture intended for publication. However, page numbers are largely missing here as well.

Folder 15 from Box 4, “Notes and drawings: Learning notes,” consists of 70 pages. Folder 15 contains very little typewritten text; most of it is handwritten. It consists mainly of shorter notes ranging from 1 to 3 pages, some of which are paginated. The dating in this folder is unclear, as all dates are missing. In general, this folder appears older than Folders 13 and 14, as Lashley here focuses heavily on Pavlov and his mentor Watson. Unlike Folders 13 and 14, there are no references to the gestalt theorists or Hull, whose debates did not become relevant until the late 1930s.

These manuscripts from the Lashley Archive thus primarily represent reflections at the end of Lashley's academic career. The psychobiologist Lashley looks back on various debates and trends, placing them in the context of his own perspective. In doing so, he also defines his own position after a quarter‐century of scientific research.

## 2 Lashley's Research in the Scientific Community

Karl S. Lashley (1958) was significant not only as a researcher but also as a central figure in twentieth‐century psychology. He was John B. Watson's most promising student at Johns Hopkins University. From 1935 to 1955 he was a professor of neuropsychological research at Harvard. Starting in 1942, he headed the Yerkes Laboratory of Primate Biology (now called the Emory National Primate Research Center), which was established in 1930 by the zoologist Robert M. Yerkes (Dewsbury 2006). In 1929, he became president of the American Psychological Association, and, a year later, was inducted into the National Academy of Sciences (NAS). In 1932, Lashley was elected to the American Academy of Arts and Sciences, and, in 1951, he became a foreign member of the Royal Society.

From the 1920s onwards, Lashley combined behaviorist experiments on animal brain function with concepts from gestalt theory and holistic thinking. When considered in conjunction, these two approaches suggest that Lashley did not seek to construct a systematic doctrinal synthesis of the two schools. Rather, he selectively appropriated specific problems and methodological approaches from each while rejecting their respective comprehensive theoretical frameworks. From gestalt psychology, he adopted an emphasis on organization, contextual relations, and the inadequacy of a purely atomistic stimulus–response model, while remaining critical of central aspects of gestalt field physiology. At the same time, he retained experimental and materialist commitments characteristic of behaviorism but rejected reductive accounts of complex behavior in terms of simple chains of reflex responses. Thus, Lashley's position is better understood as a selective engagement with, and critical reconfiguration of, elements from both traditions than as an attempt to reconcile them within a unified theoretical system.

Although Lashley's research career appears highly interdisciplinary, he consistently adhered to a materialist position and regarded himself (at least internally) as the last remaining behaviorist of the Watsonian school. Therefore, Lashley integrated gestalt theory approaches, but also criticized them on the basis of his materialistic monism when he wrote that “proponents of gestalt and continuity theories have described discriminative reactions as involving the perceptions of relations, or as based on relations between the stimuli. The implications of such statements are obscure. To say that animal reacts to a relation between stimuli is, of course, nonsense, because a relation is not a stimulus object.” (Lashley, “Learning Theory,” Box 3, Folder 13, n. p.)

In his work, Lashley brought together clinical research, ethology, and animal physiology (Dewsbury 2002a, 2002b; Weidman 1999, 2002). Notably, Lashley's openness to gestalt theory stemmed from his use of “phenomenological” methods, which he contrasted with metaphysical and subjective philosophy as early as the 1920s (cf. Lashley 1923, 347). By merging holistic gestalt psychology with functional behaviorism, Lashley, in contrast to his mentor John B. Watson, argued that the brain operates as a systemic whole. Unlike the behaviorist view of the brain as a machine, Lashley saw the brain as plastic and adaptable, describing it as a “conscious machine” (Lashley 1923, p. 330; O'Donnell 1985; Heims 1975). Lashley argued that the brain could compensate for the loss of certain areas by reallocating functions, as it operates as an integrated whole which was more than the sum of its parts. “Learning” played a crucial role in Lashley's reasoning. He argued that learning achievements were not only stored in one part of the brain. Once learned, information was not erased when part of the brain was destroyed (Lashley 1918).

In his research beginning in the 1910s, Lashley was particularly concerned with the question of whether the brain could be divided into regions and whether each of these regions could be attributed a specific function or ability. He sought to refute the still widespread localization theory of memory and learning. (Lashley 1930, 267; cf. Guenther 2015; Harrington 2009) Lashley's only monograph, *Brain Mechanisms and Intelligence*, published in 1929, developed his two central principles of “equipotentiality” based on his neurological research: according to these principles, every area of the cerebral cortex could function just as well as any other area when it came to learning and remembering. In connection with this, Lashley also developed the “Law of Mass Action,” according to which “[a]fter injuries to the brain, the rate of formation of some habits is directly proportional to the extent of injury and independent of the position within any part of the cortex“ (Lashley 1930, 271; Lashley 1929, 186). Minor interventions therefore did not significantly impair learning. They could be substituted. However, the more severe the brain injury, the greater the discrepancy between learning simple and complex habits. “This shows that the rate of learning is not dependent upon the properties of individual cells but is somehow a function of the total mass of tissue” (Lashley 1930, 271).

Beyond neural atomism, Lashley believed that the “whole” brain learned (Lashley “Learning Theory,” Box 3, Folder 13, Folder 14, n. p.). Lashley linked learning behavior and brain performance, so that for him the only explanation for behavioral changes or learning abilities was to be found in the brain (Gruevska 2022).^1^

As discussed above, Lashley was not merely a detached observer of the theoretical controversies. Through his lesion experiments, his systematic critique of associationist theories, and his empirical opposition to gestalt field‐theoretical interpretations, he actively engaged in and contributed to the ongoing theoretical debates. So, when a researcher like Lashley criticized psychological theories—as will be shown in the next step—he does not do so from a position removed from research or with disciplinary detachment, but rather as a central member of the community.

Therefore, the next step will be to show that Lashley was not actually concerned with a total rejection of theory, but rather with a “redefinition of problems” based on his materialistic monism.

## 3 Redefining the Problems of Learning

### 3.1 Lashley's Critique of Closed Theoretical Systems

Although Lashley liked to refer to his neurological data and generalize it for his concepts, from the mid‐1920s onward, he knew and shared gestalt theory's criticism of behaviorism (e.g., on the question of the unity of consciousness or regarding to localization theory) (Lashley 1923, 329; Weidman 1994, 167). Lashley was therefore aware during his investigations that there was considerable scope for interpretation of data obtained from animal experiments. “Since there is good evidence from studies with human subjects that learning may occur even with “subthreshold” stimuli, this seems to be a reasonable interpretation of the data. It is certainly not discordant with non‐continuity theory” (Lashley “Learning Theory,” Box 3, Folder 13, 7; cf. O'Donnell 1985, 59, 83, 235). The ethologist and primatologist Robert M. Yerkes had already noticed this problem in his work on the dancing mouse (Yerkes 1907). In a letter to Lashley dated February 15, 1933, he offered an ‘adverse criticism,’ despite otherwise praising Lashley's work. Yerkes emphasized that different animals could exhibit different responses to the same stimuli. He urged Lashley to limit his generalizations to rats until other species had been studied more thoroughly: “my suspicion is that you should have limited your statement to the rat until the mouse types have been studied functionally by improved methods and studied much more thoroughly in their morphology” (Robert M. Yerkes to Karl S. Lashley, 02/15/1933; cf. Rader 2022; Lynch 1988). Lashley had to agree with Yerkes’ “warning” that not all laboratory animals always reacted in the same way: “I often catch myself making this kind of generalization, and sometimes fail to catch the error, as in this case.” (Karl S. Lashley to Robert M. Yerkes, 03/05/1933).

From his engagement with gestalt theory, Lashley knew, it was always about the total situation, which represented more than just a stimulus input. The external stimulus encountered an individualized subject, who in turn selected the external stimuli internally (through attention, drives, will, intention, etc.) and therefore did not react to them immediately. (Lashley “Learning Theory,” Box 3, Folder 13, p. 3) The power of external stimuli was not unbroken, as in classical behaviorism. Internal processes also took place that had to be considered. The brain functioned as a selection and transformation system for external stimuli. Lashley recognized that there were processes in the brain that eventually manifested themselves in behavior but could not be observed directly after a stimulus. It was not easy to find an explanation for this, as neither animals nor human learning subjects (despite “verbal reports” or introspection) provided “sufficient information” for a “satisfactory interpretation of their behavior” (Lashley “Learning Theory,” Box 3, Folder 14, p. 14). Lashley regarded gestalt theory as “wholly unintelligible” to solve this problem (Karl S. Lashley to Robert M. Yerkes, 11/23/1939). At the same time, he argued that Watson's behaviorism also failed to establish a coherent terminology and continued to rely on elements of “subjective psychology” (Lashley 1923, 349). With the language of the “humanistic” system, however, it would not be possible to break down the outdated system. “Our current psychological language,” as Lashley already stated in 1923, “is a weird composite of teleological and mechanistic terms; names for phenomena which, as experienced, reveal neither purpose nor cause. The result is that a scientific description of many phenomena may not be recognized by those who are less familiar with the phenomena than with the names and their interpretative implications” (Lashley 1923, 349).

The problem with reflexologists and behaviorists was that they were too quick to separate everything subjective from the experiment, thereby losing much of the potential for explanation (Samelson 1981). In his criticism of the simplification of experimental data, Lashley therefore also turned against Pavlov and his mentor Watson (Lashley “Learning Theory,” Box 3, Folder 14, p. 6). Even individual stimuli were more complex than could be assumed from a monocausal stimulus‐response causality; rather, there was a “wide range of stimul[i]” (Lashley, “Notes and Drawings,” “Learning Notes,” Box 4, Folder 15, p. 22). Where Pavlov, at the end of the 19th century, reduced “the study of behavior to the status of a branch of physiology,” Watson was aware, in the 1910s, of the adequacy of an objective method and methodically opposed introspection. Watson's biased behaviorism was therefore more of “a denial of the claim to esoteric knowledge derived from introspection” (Lashley “Learning Theory,” Box 3, Folder 14, p. 2). The problem with multi‐layered stimuli in living organisms was precisely that they could not be traced back to subjective learning in a monocausal manner.

According to Lashley the “conditioned‐reflex theory has dealt almost exclusively with overt behavior, disregarding subjective data, even as represented in verbal reports. It has dealt also only with behavior which could be expressed quantitatively and has largely excluded inferences from qualitative differences in behavior which could not be quantified. It has avoided any significant reference to physiological processes, either as a constructive part of the theory or as limited its assumptions” (Lashley “Learning Theory,” Box 3, Folder 14, p. 1). These restrictions were, Lashley admits, partly understandable in light of a theoretical history (pure reference to reflex arcs in muscle stimulation) in the 1910s and the early 1920s, “but they are no longer justified as they were when the theory was first being developed” (Lashley “Learning Theory,” Box 3, Folder 14, p. 1). Especially when it came to synaptic processes, such a “telephone system” theory was untenable (Lashley 1930, 282). For Lashley, Watson and other behaviorists' insistence on separating physiology and psychology was justified, at the beginning of the 20th century, since objective psychology first needed to distance itself from internal, esoteric discourses like vitalism and mysticism (Lashley 1930, 273–274). When behaviorism simplified its approaches at the outset as a movement seeking to distinguish itself, this was understandable to Lashley, but this oversimplification had become a problem since the 1920s (Lashley, “Notes and Drawings,” “Learning Notes,” Box 4, Folder 15, p. 18; Lashley 1930, 273).

American psychology students had long followed in Watson's footsteps by striving for an objective science that separated behavioral research (in the sense of psychology of internal processes) from physiology. However, this separation of the “study of behavior” and physiology had become problematic. It would have to be overcome with neuropsychology. New “anatomical and physiological studies” in the 1930s and 1940s, including those by Spanish neuroscientist Rafael Lorente de Nó, “demonstrated a possible mechanism for sustained central‐nervous action with unlimited elaboration before any overt activity is produced. Electrophysiology provides supporting evidence for such central activity and some possibility of its direct analysis” (Lashley “Learning Theory,” Box 3, Folder 14, p. 2. cf. Lorente De Nó 1938a; Lorente de No 1938b). It was not the better argument or the more convincing theory, but rather more accurate (brain) research and larger data collection that prompted a rethink. New brain research in conjunction with new technical equipment such as the EEG enabled physiological, quantifiable access to the inner self, which behaviorism had always denied and, as Lashley was able to show, continued to deny, due to its (reflexology) method and static machine metaphors (Borck 2018). Even though the approach and quantification were similar to the analysis‐control paradigm, behaviorism was unable to integrate the inner, mental‐cognitive aspect into its rigid concept.

With Lashley's emphasis on the central role of the brain, the subjective was given the same status as external reactions. This meant that internal and external behavior, although differing in complexity, were equally experimentally measurable and physically plastic (Lashley 1923, 344). “Critical examination of “subjective” phenomena has reduced them to the same status as overt reactions; both may be regarded as imperfect indicators to the integrative activities of the brain. There is no present reason to sustain the doctrines of methodological behaviorism, with respect to disregarding introspective data. Verbal report of experience given evidence of central‐nervous activities which cannot at present be obtained by any other method” (Lashley “Learning Theory,” Box 3, Folder 14, p. 2). The behaviorist critique of introspection came to nothing, because introspection as subjectification could also be explained neurologically. Learning processes could be interpreted physiologically via the brain, as Lashley attempted to show with his own studies on rats that had undergone brain surgery. “The construction of learning theories without reference to physiology seems also a needless and unprofitable limitation” (Lashley “Learning Theory,” Box 3, Folder 14, p. 3).

If materialistic psychologists such as Watson were already too one‐sided in restricting their observations, then gestalt theorists were even stricter—and they also went in the wrong direction with their restrictions. This is because gestalt psychology's conclusions were based almost exclusively on introspection or what it considered “meaningful distortions of reproductive memory” (Lashley “Learning Theory,” Box 3, Folder 14, p. 6). Neither gestalt theorists nor objectivist psychologists have managed to harmonize their theories “with data obtained by the other method. Koffka mentions very few quantitative studies and dismisses the results as of little importance for his theory, without attempting to show how they might have been derived from his assumptions” (Lashley “Learning Theory,” Box 3, Folder 14, p. 6).^2^ Thus, the subjective and objective descriptions were “not descriptions from two essentially different points of view, or descriptions of two different aspects, but simply descriptions of the same thing with different degrees of accuracy and detail” (Lashley 1923, 338). The incompatibility stemmed not from the object itself, but from the approach taken to study it.

In Lashley's view, the “complete insularity” of the two theoretical approaches was evident in the fact that, for example, of the 400 references cited by opponents Clark Hull and Kurt Koffka, only one was cited by both, and this was used by both as evidence for their own theory (Lashley, “Learning Theory,” Box 3, Folder 14, p. 7). “The difference in fundamental point of view appears to be so profound that neither finds any significance in the major evidence upon which the other bases his study. In such a situation one must raise the question, what kinds of data are suitable as a starting point for the development of learning theory: By what criteria are they to be selected?” (Lashley, “Learning Theory,” Box 3, Folder 14, p. 7).

For Lashley's fundamental criticism, the different learning concepts were determined not by the experimental data alone, but by a “background theory” (Lashley “Learning Theory,” Box 3, Folder 14, n. p.). “Learning” could only be interpreted via another psychological system upon which it was formulated. A psychological system that stabilized itself through the appropriate experimental systems. “I [Lashley] have also stated that when optimal conditions for learning are present learning may be very rapid, even reduced to one trial, but I do not believe it possible now to specify the optimal conditions” (Lashley “Learning Theory,” Box 3, Folder 13, n. p.). Consequently, if something could only be discovered under optimal conditions, the experimental conditions became fundamental for the “formulation of any learning theories.” In Lashley's view, the conflicting theories of continuity and non‐continuity theory had clarified some points but left others “confused.” The continuity theory was “allied to the more general Pavlovian theory of behavior” (Lashley “Learning Theory,” Box 3, Folder 13, n. p., cf. McCulloch and Pratt 1934). The non‐continuity theory was based on gestalt psychology.

As a founding myth of learning theories, Lashley cited Hermann Ebbinghaus’ influential learning curves, which were a major “inspiration” for later learning theories (Lashley “Learning Theory,” Box 3, Folder 14, n. p.; Murray 2021). The learning curves were used, among other things, to show that “learning is a swelling of the neuron and that it is an autocatalytic monomolecular reversible chemical reaction” (Lashley “Learning Theory,” Box 3, Folder 14, n. p.). Lashley was critical of this interpretation, because he wanted to distinguish between “learning how and remembering” (Lashley “Learning Theory,” Box 3, Folder 14, n. p.). The singular data points on the learning curve would be far too individualized. What you would need is a large number of learning curves, which you would then superimpose on top of each other to obtain an average value. But even then, according to Lashley, there was the problem of adjusting the curves, which would conceal their significance: “the smoothing of learning curves may be chiefly a device for concealing their meaning” (Lashley “Learning Theory,” Box 3, Folder 14, n.p.). The adjusted data did not reflect the actual experimental situation. It was not the adjusted curve (“smooth curve”) that was significant for understanding what happened during learning, but rather the “irregularities” of the curve (Lashley “Learning Theory,” Box 3, Folder 14, p. 8). For Lashley, the learning curve was „ambiguous” (Lashley “Learning Theory,” Box 3, Folder 14, p. 8).

In Lashley's view, data, concepts, or terms in learning theories should be treated with caution. This is because many of them were biased (by researchers and experimental apparatus). Pavlov's neurological theories were “inadequate and the continued use of these terms […] is therefore misleading” (Lashley “Learning Theory,” Box 3, Folder 14, n.p.; Todes 2014). Even the more thoughtful behaviorists, such as Edward C. Tolman with his “intervening variables” or the hypothesis concepts (by Isadore Krechevsky), failed to make any progress (Lashley “Learning Theory,” Box 3, Folder 14, n.p.; Hebb 1949, 6). They served only to obscure the tautological nature of the theory (Lashley “Learning Theory,” Box 3, Folder 14, n.p.).

For Lashley, the solution for broadening the perspective was data‐driven. According to Lashley's explanation, researchers who only had second‐hand knowledge of animal experiments often lacked large, valid data sets. The insufficient data was then interpreted only in terms of their own theory. This necessarily made the theory circular and fundamentally closed to other approaches. This led to an incompatibility of approaches and an epistemological stalemate. “If objective psychologists have erred in restricting their range of observations, the gestaltist group has dealt with an even more restricted range. Their conclusions have been based on almost entirely on introspective data or the meaningful distortions of reproductive memory. They have made no serious attempt to harmonize their theories with data obtained by other methods. Koffka mentions very few quantitative studies and dismisses the results as of little importance for his theory, attempting to show how they might have been derived from his assumptions” (Lashley “Learning Theory,” Box 3, Folder 14, n. p.). For the animal researcher Lashley, the problem was therefore not anthropomorphism, as many critics attributed to behaviorist and gestalt theory movements (Lashley “Learning Theory,” Box 3, Folder 14, p. 14; Wheeler et al. 1931; Dacey 2017; Kennedy 1992; Daston 2005, 37–58). Instead the vague and incomplete data provided too much scope for interpretation. “The data obtained from studies of learning animals are too incomplete to permit a valid interpretation.” (Lashley “Learning Theory,” Box 3, Folder 14, p. 14). In addition, many processes in animal research and brain research could not be directly investigated, which led to the early emergence of an apparatus science that produced polyvalent, technologized data (images) that were extremely open to interpretation (Borck 2008). The technical experimental systems, the equipment, the recording systems for graphs, curves, measurements—everything was written down in accordance with the various learning theories (De Chadarevian 1993; Frank 1994).

The sharp criticism between the different learning theories (Köhler vs. Thorndike, Tolman vs. Hull, “scientific vs. humanistic” [Lashley 1923, 346]) became understandable to Lashley when he learned to comprehend the “prejudices of the critic” (Lashley “Learning Theory, Box 3, Folder 13, n. p.). Lashley therefore saw the choice of method not as a “rational” decision, but rather as an “emotional” one. It was a question of “temperament” (Lashley 1923, 345). At the same time, however, this meant that there was no longer a single verifiable point of view; everything was always a matter of perspective.^3^

But how could a way out of this linguistic and theoretical impasse be found? How could inner learning and behavioral processes be accessed when they were so neurologically complex and defied monocausal explanation?

### 3.2 Lashley's Methodological Alternative

Lashley's “exceedingly astigmatic” (Lashley 1923, 337)^4^ approach moved away from the epistemic conflict between behaviorism and gestalt theory, which led to no solution or new insights, in order to achieve a “redefining of the problems of learning” (Lashley “Learning Theory,” Box 3, Folder 14, n.p.). Lashley's concrete epistemology led away from unified theory and grand conception and toward large amounts of data, material, and the quantified brain. This supposed departure from comprehensive psychological systems was nevertheless connected to a materialist monism (Lashley 1923, 343). Consequently, Lashley also had a “background theory,” but he did not present it as such. Instead, he tried to give the impression that his research on the brain was guided solely by data and experience. The brain was the central organ that was supposed to provide incentives for new approaches and new terminology that would reconcile the conflicting areas in the form of a neurologization of the psyche (Lashley 1930, 266). The techniques used to describe neurological processes no longer relied on automatic or electronic metaphors (machine or “telephone”) from reflex theory (Lashley 1930, 267; Lashley 1950, 454; cf. Daugman 2001, 272; Weidman 1994; Boden 2006), but instead used techno‐organicist and biologically dynamic concepts (like “plasticity,” “system,” or “network”) (Bates 2016; Hebb 1952; Heyck 2015; Killen 2023; Kline 2015; Prkachin 2026; Tamborini 2022).

In his approach, Lashley seeks to link complex intraneuronal problems (“What happens in the brain when an association is formed?”, “Must the exciting activate directly the same neurons which were altered in learning or may similar neural organizations excite the engram, without identity of neuronal elements?” (Lashley “Learning Theory,” Box 3, Folder 14, p. 3) with the question of adequate data collection, that is, methodology, rather than a specific theory. This move was particularly astute, as it enabled Lashley, the “advocatus diaboli,” to reconceptualize psychological problems as questions of neural organization (Lashley “Learning Theory,” Box 3, Folder 14, p. 4).

By shifting the focus to data and selection criteria, Lashley was concerned with information management that embedded data and viewed it within the overall system. In Lashley's interpretation, the canonized dichotomies and restrictive exclusions of behaviorism and gestalt psychology overlooked an important aspect, which was that “a large proportion of experimental studies are not scientific and […] non‐experimental observations.” (Lashley, “Learning Theory,” Box 3, Folder 14, p. 5). Simply focusing on measurement did not help. “Since Ebbinhaus, measurment has been regarded as a fundamental requirement for the scientific study of learning. But obtaining a set of numbers, presented with their appropriate chi square values, is no guarantee that the study was scientific, although it is often considered to be in bad taste to ask whether the number mean anything” (Lashley, “Learning Theory,” Box 3, Folder 14, p. 5). Although this statement may seem a surprise coming from Lashley, a researcher who believed in experimentation, he criticized pure quantification as mindless data collection without accurate observation and embedding in the overall context or, indeed, the overall organism. As previously discussed regarding learning curves, the data were open to various interpretations depending on the method used. The one‐sided focus on the experimental system had turned the “history of learning theory” into a “history of fashion in experiment”: “Memorizing nonsense syllables, motor learning, solving puzzles, logical learning, maze studies, Pavlovian conditioning, each has had its day and has produced a learning theory, biased by the limited range of data considered” (Lashley, “Learning Theory,” Box 3, Folder 14, p. 5). Each experiment had its own justification and theoretical basis. At the same time, however, each experiment evoked and reinforced further theories with its data. The series of experiments became as meaningless as Ebbinghaus’ syllable sequences. Quantification in the experiments was always associated with technologization. Apparatuses as “agents of knowledge” were used by Pavlov, Thorndike, and Watson, as well as by critics like Köhler, Tolman, and Goldstein (Wulz 2010). Lashley now clearly recognized that the apparatus had become a knowledge agent, thereby creating a dependency on what Daston & Galison describe as “mechanical objectivity” (Daston and Galison 2007). The double‐edged nature of this “mechanical objectivity” was due to the fact that “instruments and special controls” are “essential for validation of certain types of observation, but in the hands of the unobservant or of those who knows animals only at second hand, the limited data obtained under recorded under such conditions have too often represented the whole phenomenon of learning” (Lashley, “Learning Theory,” Box 3, Folder 14, p. 6).

With the supposed focus solely on experimental data, it was natural to assume a limited data set at the outset. Initially, all data was poor, and the experimenter had to try to interpret the physiological data in the first step (Lashley, “Learning Theory,” Box 3, Folder 14, p. 4). However, especially in learning experiments, there were far too many possibilities and ambiguities in data interpretation. Empirical knowledge was required that could not simply be substituted by an experimental setup. Experimentation and experience had to go hand in hand for Lashley. “It is very difficult to devise an experimental situation which can provide crucial evidence for the points at issue. In general, the more intricate the situation, the less likely it is to give clear out evidence” (Lashley, “Learning Theory,” Box 3, Folder 14, p. 7).

This simplified representation of maze learning was popular among learning researchers because it provided a more controlled environment. The maze, as a human‐made, artificial environment, led to anthropomorphic conclusions. Since, according to Lashley, the problems were not natural but human‐made, animals could only respond to them in a “human” way. The data was contaminated with “human” influences. Lashley was “convinced that that many attempts to account for maze learning have led to an oversimplification of the problem that is as harmful to scientific investigation as was the old anthropomorphism. The maze habit is an extremely complex thing and we are scarcely nearer ananalyis [sic] of the activities involved in learning it that [sic] we are to an analysis of the process of learning the calculus” (Lashley, “Notes and Drawings,” “Learning Notes,” p. 18). In addition to anthropomorphizing, the generalization mentioned above was a major problem for Lashley, as it led to stimuli and responses being viewed in an undifferentiated manner. “The reinforcement of activity is usually not general, but confined to certain types of activity, while other types are inhibited. The sexually excited rabbit may be indifferent to food, the hungry chick to companions” (Lashley, “Notes and Drawings,” “Learning Notes,” p. 22). The traditional theories of summation or amplification could not be applied here, as these theories were based on the phase relationships of nerve impulses and what we were dealing with was clearly a continuous summation. Thus, as Yerkes had already criticized, animals always responded to something different, depending on their individual (neuron‐based) situation. In all “primitive cases of learning” that did not require animal training, a stimulus tended to accumulate, “spread, and reinforce reactions to other stimuli” (Lashley, “Notes and Drawings,” “Learning Notes,” n.p.). If a stimulus (such as a visual stimulus [VS]) could stimulate several internal processes, the reaction was complex and intertwined.

Because contemporary theories imposed “artificial restrictions on learning theory,” Lashley adopted a more pragmatic approach. This approach was grounded in empirical data *and* experience (Lashley, “Learning Theory,” Box 3, Folder 14, p. 1.). “The facts of cerebral physiology are so varied, so diverse, as to suggest that for some of them each theory [behaviorism, gestalt theory] is true, for all of them every theory is false” (Lashley 1930, 283). If one wanted terminology in Lashley's sense, it had to be based on neurological and physiological data and enriched with experience. Lashley recognized that there were no simple, hasty answers to neurological problems, “but it should be possible to decide what kinds of learning data are relevant to the questions and what facts of neurology may limit the physiological theories which can be formulated. In discussions of learning theory I have always appeared as advocatus diaboli and I have no intention now of attempting to formulate a “grand” theory of memory […]. However, it should be possible by a broad survey of the phenomena of memory and of neural activity to determine what facts now seem most relevant for an understanding of the neurology of learning and what directions of research are most promising for a solution of the problems. It may be possible to translate some of the concepts of current theories of learning into neurological terms or, if not, to show that they are meaningless from such a point of view. It is this redefining of the problems of learning that I propose to attempt […]” (Lashley, “Learning Theory,” Box 3, Folder 14, n.p.).

This required a new approach to “learning.” Combining behaviorist quantification with gestalt theory's conception of the environment and a holistic approach, Lashley located learning problems in the brain (Lashley 1923, 333). Lashley developed his psychobiological approach as a neurophysiological alternative to these two opposing currents, motivated by his distrust of their theory‐driven selection and interpretation of empirical evidence. Behavior was no longer as easily visible, and therefore analyzable and controllable, as it had been in behaviorism. However, it was also not a matter of an entire qualitative‐individual organism moving in a comprehensible environment, as in gestalt theory. This is because the primacy of direct (supposedly immediate) observation, which gestalt theorists and behaviorists shared, fell away when (intra)neuronal processes came into focus. For Lashley, the third way was a conclusion drawn from the experimental destruction of brain areas. Since he had little direct evidence for the nature of neural learning processes, he had to derive laws from the effects of brain injuries. It was interventions into the brain itself that provided the basis for theory, rather than observations of outward learning behaviors.

Lashley's new questions were formulated as follows: “My own interest in learning is in the internal phenomena. What happens in the brain when something is learned? What is the neural organization in perception, recognition, recall? How are the effects of this organization transmitted to the motor system? The external relations revealed by behavior studies of learning are significant except as contributions to human engineering, only in so far as they contribute to an understanding of the intervening neural processes” (Lashley, “Learning Theory,” Box 3, Folder 14, p. 3).

When Lashley approached the brain as an integrated whole, in accordance with his commitment to materialist and neurophysiological explanations, he was not concerned with separating behaviorism and gestalt theory, physiology and psychology, but rather with separating external and internal phenomena (Lashley 1923, 334). From his perspective, separating psychology from physiology was a “dangerous practice” (Lashley, “Learning Theory,” Box 3, Folder 14, p. 4). “There is no real distinction in problems of methods between the two disciplines. The contention that physiology deals with separate organs whereas psychology deals with the whole organism is fallacious. No psychologist has ever dealt with the whole organism. He dissects it into functions and studies them separately and it is his misfortune that he cannot isolate them as completely as the physiologists can isolate organs. And physiologists often deal with the integration of functions far more adequately than the psychologist has as yet succeeded in doing. The distinction, if any is now justified, seems to be between the study of external phenomena versus internal. The restriction of the study of learning to the former class puts it under a quite unnecessary handicap” (Lashley, “Learning Theory,” Box 3, Folder 14, p. 4).^5^

## 4 No Escape From Theory

This article has shown that Lashley's importance lies not in a grand theory of learning, but in his epistemological critique of theory‐driven interpretation itself (Weidman 1994, 164; 1996). By highlighting the power of “background theories” in shaping experimental meaning, Lashley exposed the limits of both behaviorism and gestalt psychology without attempting their synthesis. With his criticism of behaviorism and gestalt theory, Lashley attempts to hide his own bias (materialistic monism). By focusing purely on data and experience with the experimental animal, Lashley's approach appears objective and serious.

The archival material therefore showed that Lashley's achievement did not consist in escaping theory through data. Rather, the evidence supports a subtler interpretation: Lashley became skeptical of comprehensive psychological systems because they predetermined what could count as admissible evidence, while he continued to pursue materially grounded explanations of neural organization. His contribution thus lay less in rejecting theory than in redefining the conditions under which theoretical claims should be constrained by heterogeneous forms of behavioral, experimental, and neurophysiological evidence.

## Author Contributions

The author was solely responsible for all aspects of the study and article preparation and approved the final version of the article.

## Data Availability

The data that support the findings of this study are available from the corresponding author upon reasonable request.

## Endnotes

1 On the problematic racist foundations of Lashley's anthropology, which, according to Weidman, also influenced his epistemology cf. Weidman 1999. The research paper is funded by the Deutsche Forschungsgemeinschaft (DFG, German Research Foundation) – Project number 492533313

2 With reference to behaviorism and introspection, Lashley states that “behaviorism presents the possibility of a more nearly complete analysis of the same data.” (Lashley 1923, 338).

3 Lashley already put forward this argument in a defense of behaviorist consciousness research against a “subjective science” (cf. Lashley 1923, 337). On the inconsistency of learning research in the 1930s, which was often understood as a “crisis” (cf. Langer 1936; Langer 1937). Lashley sounds very much like Thomas S. Kuhn and his “paradigms” here (Kuhn 1962), even though Lashley predates him by several decades. This similarity might stem from their shared engagement with Gestalt psychology.

4 To Lashley's anti‐theoretical view cf. Weidman, 1994, 163‐164.

5 Genetics served as the model for Lashley's interdisciplinary research, cf. Lashley to John B. Watson, 04/12/1957; Lashley, “Learning Theory,” Box 3, Folder 14, p. 4.
